# Mutations in the PCNA-binding site of CDKN1C inhibit cell proliferation by impairing the entry into S phase

**DOI:** 10.1186/s13008-015-0008-8

**Published:** 2015-03-28

**Authors:** Kleiton S Borges, Valerie A Arboleda, Eric Vilain

**Affiliations:** Department of Human Genetics, David Geffen School of Medicine at UCLA, University of California, Los Angeles, 695 Charles E. Young Drive, Los Angeles, CA 90095 USA; Department of Genetics, Ribeirão Preto Medical School, University of São, Ribeirão Preto, Av. Bandeirantes 3900, CEP 14049-900 Ribeirão Preto, SP Brazil; Department of Pathology and Laboratory Medicine, David Geffen School of Medicine at UCLA, University of California, Los Angeles, USA; Department of Pediatrics, David Geffen School of Medicine, University of California, Los Angeles, USA; Department of Urology, David Geffen School of Medicine, University of California, Los Angeles, USA

**Keywords:** Cell cycle, CDK-inhibitor, IMAGe syndrome, Cyclin, Beckwith Wiedemann syndrome, Intrauterine growth restriction

## Abstract

*CDKN1C* (also known as *P57*^*kip2*^) is a cyclin-dependent kinase inhibitor that functions as a negative regulator of cell proliferation through G1 phase cell cycle arrest. Recently, our group described gain-of-function mutations in the PCNA-binding site of *CDKN1C* that result in an undergrowth syndrome called IMAGe Syndrome (*I*ntrauterine Growth Restriction, *M*etaphyseal dysplasia, *A*drenal hypoplasia, and *Ge*nital anomalies), with life-threatening consequences. Loss-of-function mutations in *CDKN1C* have been identified in 5-10% of individuals with Beckwith-Wiedemann syndrome (BWS), an overgrowth disorder with features that are the opposite of IMAGe syndrome. Here, we investigate the effects of IMAGe-associated mutations on protein stability, cell cycle progression and cell proliferation. Mutations in the PCNA-binding site of *CDKN1C* significantly increase CDKN1C protein stability and prevent cell cycle progression into the S phase. Overexpression of either wild-type or BWS-mutant CDKN1C inhibited cell proliferation. However, the IMAGe-mutant CDKN1C protein decreased cell growth significantly more than both the wild-type or BWS protein. These findings bring new insights into the molecular events underlying IMAGe syndrome.

## Findings

CDKN1C (also known as p57^kip2^) is a cyclin-dependent kinase inhibitor sharing CDKN1B (p27^Kip1^) and CDKN1A (p21^WAF1/Cip1^) homology that has a key role in inhibiting cell cycle progression by blocking the cells in G1 phase [1]. It is located on chromosome 11p15.5 and is part of a cluster of imprinted genes. The CDKN1C protein has 316 amino acids (AA) and consists of three structurally distinct domains: the N-terminal domain (AA 1 – 110), necessary for CDK inhibition; a central proline-alanine series of repeats (PAPA-repeats, AA 156 – 213), and a highly conserved C-terminal region (QT domain, AA 214 – 316), where the PCNA binding site is located [2,3]. In nearly all tissues, *CDKN1C* is expressed from the maternal allele while the paternal allele is silenced [1,2].

Recently, our group identified missense mutations in the PCNA-binding domain of *CDKN1C* in families with IMAGe syndrome (MIM# 614732), characterized by *I*ntrauterine growth retardation, *M*etaphyseal dysplasia, *A*drenal hypoplasia and *Ge*nital anomalies.[4] Interestingly, *CDKN1C* mutations have previously been identified in Beckwith-Wiedemann Syndrome (BWS; MIM# 130650) and are distributed throughout entire length of the coding sequence of the gene. These BWS-associated mutations are either missense mutations that disrupt the cyclin-dependent kinase binding domain, or nonsense mutations, both of which result in protein loss-of-function [3,4]. *CDKN1C* mutations have been found in 5–10% of sporadic BWS cases and in approximately 40% of cases with a family history [3].

We hypothesize that IMAGe syndrome mutations result in loss of PCNA-binding and a gain-of-function of CDKN1C. Previous studies demonstrated that IMAGe-mutant CDKN1C and BWS-mutant CDKN1C have differential effects on cell cycle progression, developmental processes, suggesting that domain-specific mutations act through distinct mechanisms [4]. Early studies have also shown that targeted eye expression of IMAGe-mutant *CDKN1C* mutations in *Drosophila* cause severe growth defects compared to wild type *CDKN1C.* Furthermore, an *in vitro* assay showed that IMAGe-mutant CDKN1C protein presents alteration in its ubiquitination pattern [4]. Here, we aim to investigate the molecular mechanisms underlying the IMAGe mutations. To address this, we performed functional studies with wild-type, IMAGe-mutant and BWS-mutant CDKN1C proteins. IMAGe-mutant protein consists of a mutation in the PCNA binding domain and an intact cyclin-dependent kinase binding domain whereas CDKN1C BWS-mutant protein contains a mutation in the cyclin-dependent kinase binding domain and a wild-type PCNA binding site. Our results showed that missense mutations in the PCNA-binding site of CDKN1C lead to protein stabilization, impaired entry into S-phase, culminating in decreased cell proliferation. Moreover, IMAGe-mutants displayed different effects relative to BWS-mutants, suggesting that domain-specific mutations have differential effects on cell-cycle progression and cell proliferation.

### Loss of PCNA binding in IMAGe-mutant *CDKN1C* results in increased protein stability and loss of normal oscillation of CDKN1C protein during cell cycle progression

**Figure 1:**
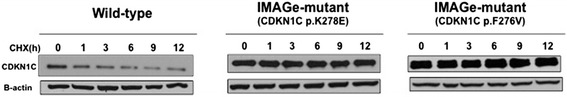
**IMAGe-mutant CDKN1C stabilizes CDKN1C protein.** Cells were transiently transfected with plasmids expressing CDKN1C wild-type or IMAGE-mutants (p.K278E and p.F276V). After addition of cycloheximide (CHX) at 100 μg/mL to the culture medium, cells were harvested at various times and processed by Western blot analysis of CDKN1C protein.

Mutations in the PCNA binding domain of the related protein CDKN1A, which shares sequence homology with CDKN1C, prevents its degradation [5]. Therefore, we decided to investigate whether the loss of direct binding of PCNA alters the degradation of CDKN1C. To address this, we transiently overexpressed wild-type or IMAGe-mutant (p.K278E and p.F276V) *CDKN1C* into HEK293T cells and examined the protein degradation rate of CDKN1C in the presence of the *de novo* protein synthesis inhibitor cycloheximide (100 μg/mL). pcDNA3.1-FLAG-CDKN1C constructs used here were described previously [4]. The following antibodies were used in our western blot experiments: anti-CDKN1C (Santa Cruz Biotechnology, sc-1040), anti-Cyclin A (Santa Cruz Biotechnology, sc-271682) and anti-B-actin (Abcam, mAbcam 8226). The secondary antibodies used were goat anti-mouse HRP (Bio-rad, 170–5047) and goat anti-rabbit HRP (Santa Cruz Biotechnology, sc-2030). IMAGe-mutant CDKN1C (p.K278E and p.276 V) led to increased stabilization of CDKN1C compared to wild-type CDKN1C (Figure 1). This is in accordance with recently published results, where IMAGE-associated proteins also demonstrated increased stability compared to wild-type [6,7].

**Figure 2:**
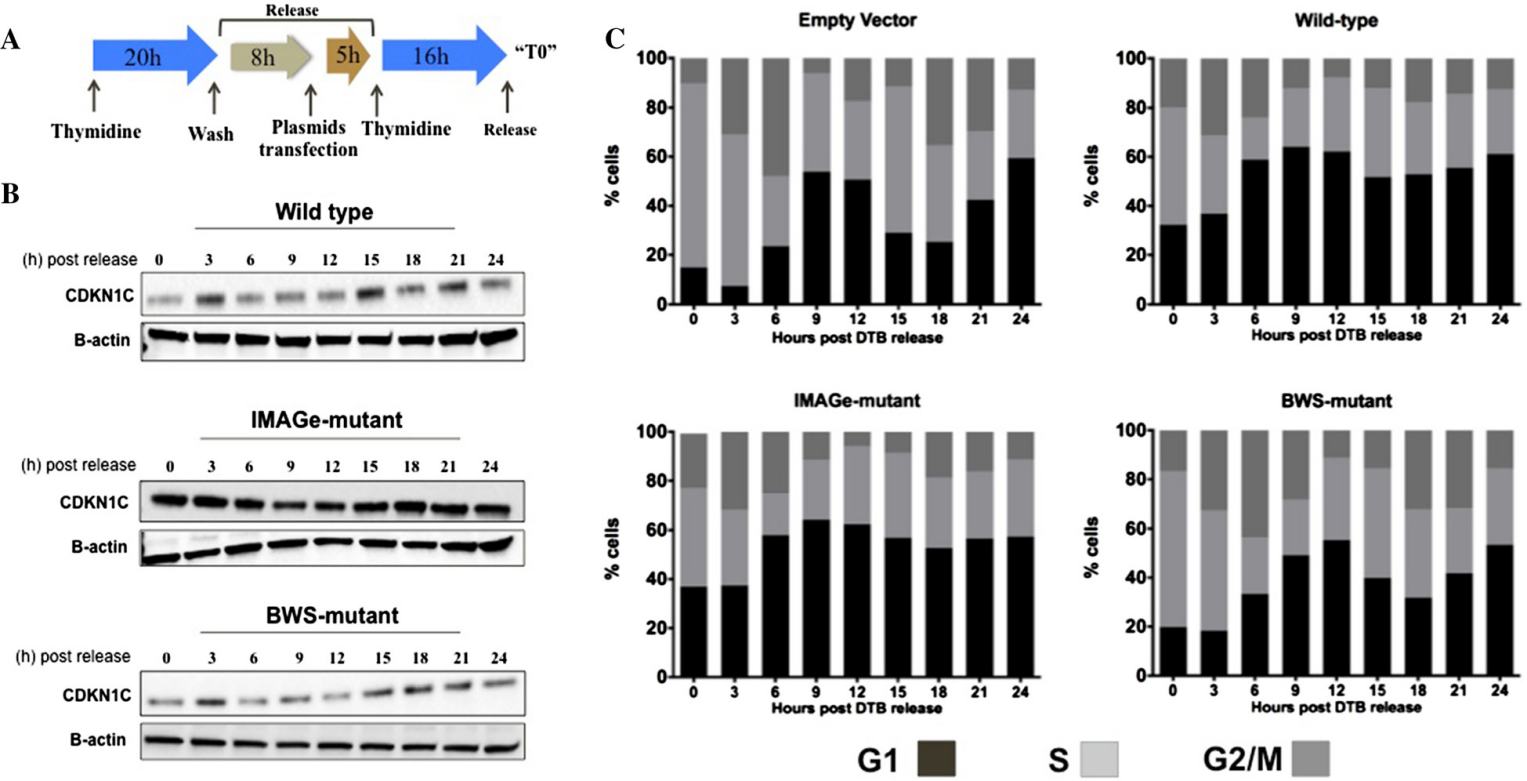
**Synchronized cells transfected with IMAGe-mutant CDKN1C show loss of oscillation of CDKN1C through the cell cycle. (A)** Experimental outline for synchronization of HEK293T cells. Cells were synchronized with double thymidine block as on indicated. After the first block cells were transfected with a empty, wild-type CDKN1C, BWS-mutant CDKN1C (p.L42P) or IMAGE-mutant CDKN1C (p.K278E) and then synchronized again in early S phase with thymidine. After this process, a time was designated as “T0” and the cells were released and progressed through the S-phase **(B)** Upon release, cells were collected at the indicated times and CDKN1C was analyzed by immunoblotting. Beta-actin was used as a loading control. **(C)** Cell cycle analysis of double thymidine block synchronized cells transfected with empty plasmid, wild-type CDKN1C, BWS-mutant (p.L42P) or IMAGE-mutant (p.K278E) CDKN1C.

Regulation of protein turnover is required for normal progression through the cell cycle. Since loss of PCNA-binding led to increased stabilization of CDKN1C, we hypothesized that the turnover of CDKN1C during the cell cycle progression might be altered. To investigate this, we developed a protocol where HEK293T cells progress into S-phase in the presence of different CDKN1C proteins (wild-type, IMAGe-mutant (p.K278E) and BWS-mutant (p.L42P)). The BWS-mutant has a mutation in the cyclin dependent kinase (CDK) inhibitory domain, but expresses a normal PCNA binding domain, while the IMAGe-mutant p.K278E has been previously shown to have loss of PCNA binding [4]. Cultures of HEK293T cells were synchronized using an initial thymidine block (2 mM) at G1/S border phase and upon release of the thymidine block transfected with their respective plasmids. Cells were resynchronized with thymidine (Figure 2A) and after a second wash were harvested at various time points and processed for western blot and cell cycle analysis, as described [4].

Western blot analysis showed that in cells transfected with either the wild-type or BWS-mutant CDKN1C, the CDKN1C protein levels oscillated, peaking at 3 and 15 hours after the release. On the other hand, protein levels of IMAGe-mutant CDKN1C remained at constant levels throughout the entire 24-hour time course (Figure 2B). These results show that IMAGe mutations result in increased stability and a loss of normal oscillation of CDKN1C protein levels. This is consistent with previous results showing loss of ubiquitination [4] and indicates that normal progression through the cell cycle is disturbed when the IMAGe-mutant is expressed.

### IMAGe-mutant CDKN1C impairs entry into S phase in synchronized cells

In order to understand the effects of CDKN1C on cell cycle progression, we examined cell cycle phase distribution of the cells from the experiment described in Figure 2A. Upon release at G1/S phase, cells transfected with empty plasmid or BWS-mutant CDKN1C progressed synchronously through the entire cell cycle (Figure 2C) whereas a larger proportion of wild-type and IMAGe-mutant CDKN1C transfected cells were unable to progress through the cell cycle and remained in G1 phase after T0 (Figure 2C). These data indicate that IMAGe-mutant CDKN1C protein prevents G1/S phase transition as CDKN1C wild-type protein.

**Figure 3:**
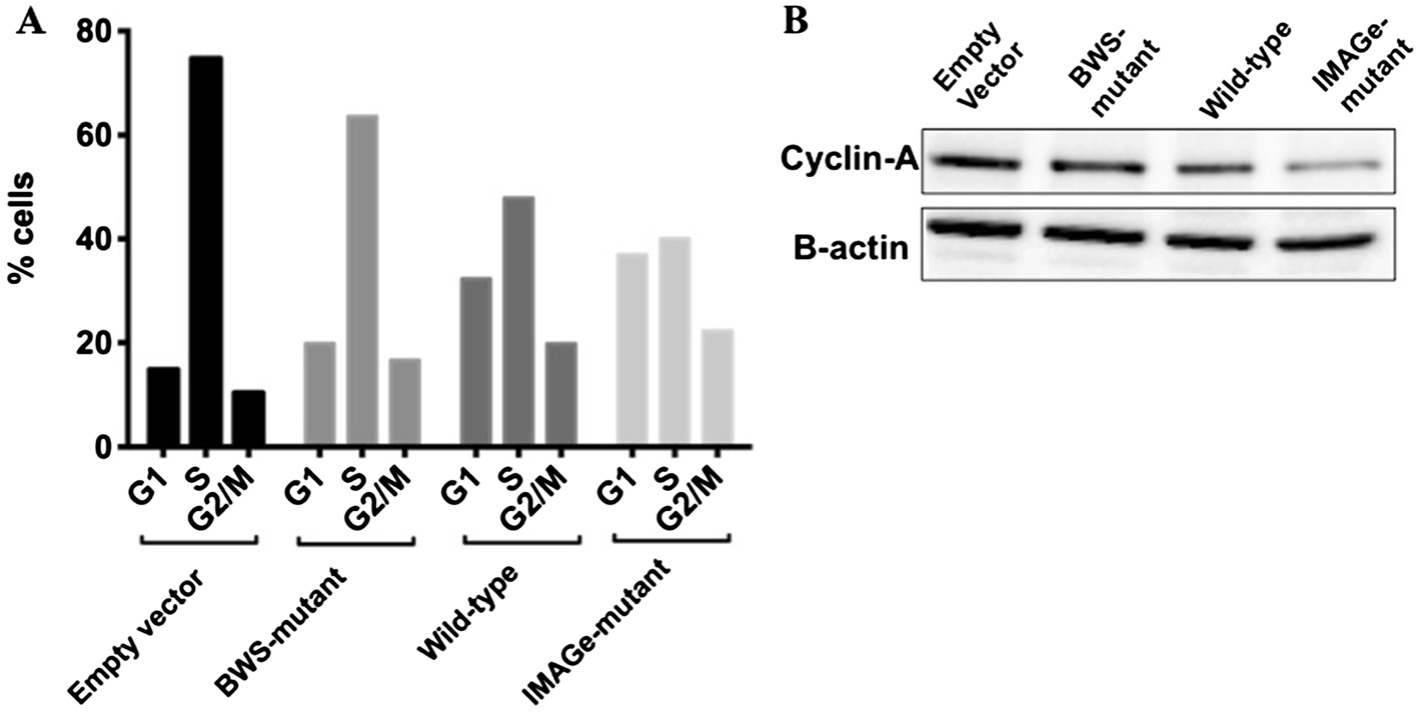
**IMAGe-mutants have decreased progression through S-phase after synchronization in S-phase. (A)** Cell cycle analyses by flow cytometry of cells at T0 transfected as described in Figure 2A. **(B)** Immunoblot analysis of transfected cells showed a failure of accumulation of Cyclin-A, an S phase marker in IMAGe-mutant transfected cells at time T0.

To further investigate this, we examined cell cycle phase distribution of the cells at time T0. The percentage of the cells in the G1 phase was increased by the transfection of wild-type CDKN1C compared with empty plasmid and BWS-mutant (32.31%, 14.88% and 19.85% respectively). However, cells transfected with IMAGe-mutant CDKN1C had the highest percentage of cells in the G1 phase (36.94%) and the lowest proportion of cells in the S-phase (40.06%) (Figure 3A).

Given the differences observed between wild-type, IMAGe-mutant and BWS-mutant CDKN1C in the G1 and S-phase cell distributions, we also examined levels of cell cycle regulatory protein cyclin-A, a well established S-phase marker [8]. Protein lysates from cells transfected with IMAGe-mutant CDKN1C did not exhibit accumulation of cyclin A as observed in cells transfected with empty vector, wild-type, and BWS-mutant CDKN1C (Figure 3B). This data shows that the loss of S-phase transition occurs in cells transfected with IMAGe-mutant CDKN1C, but not in empty vector, wild-type, or BWS-mutant CDKN1C transfected cells. In accordance with our results, Brioude et al., [6] found a higher percentage of cells in G1 phase in asynchronous cells transfected with IMAGe-mutant. Collectively, these findings show that the increased CDKN1C stability conferred by mutations in the PCNA-binding domain mutations leads to impaired entry into S phase.

### IMAGe-mutant CDKN1C decreases cell proliferation

To investigate the effects of CDKN1C protein stability on cell proliferation we performed a clonogenic assay with the wild-type, BWS-mutant and IMAGe-mutant CDKN1C in HEK293T and SW13 cell lines. After transfection cell suspensions were split into six-well plates. The cell cultures were incubated for 7 days and the colonies were then rinsed with PBS, fixed with methanol, and stained with Crystal violet stain. Colonies with more than 50 cells were counted. Assays were performed in triplicate.

**Figure 4:**
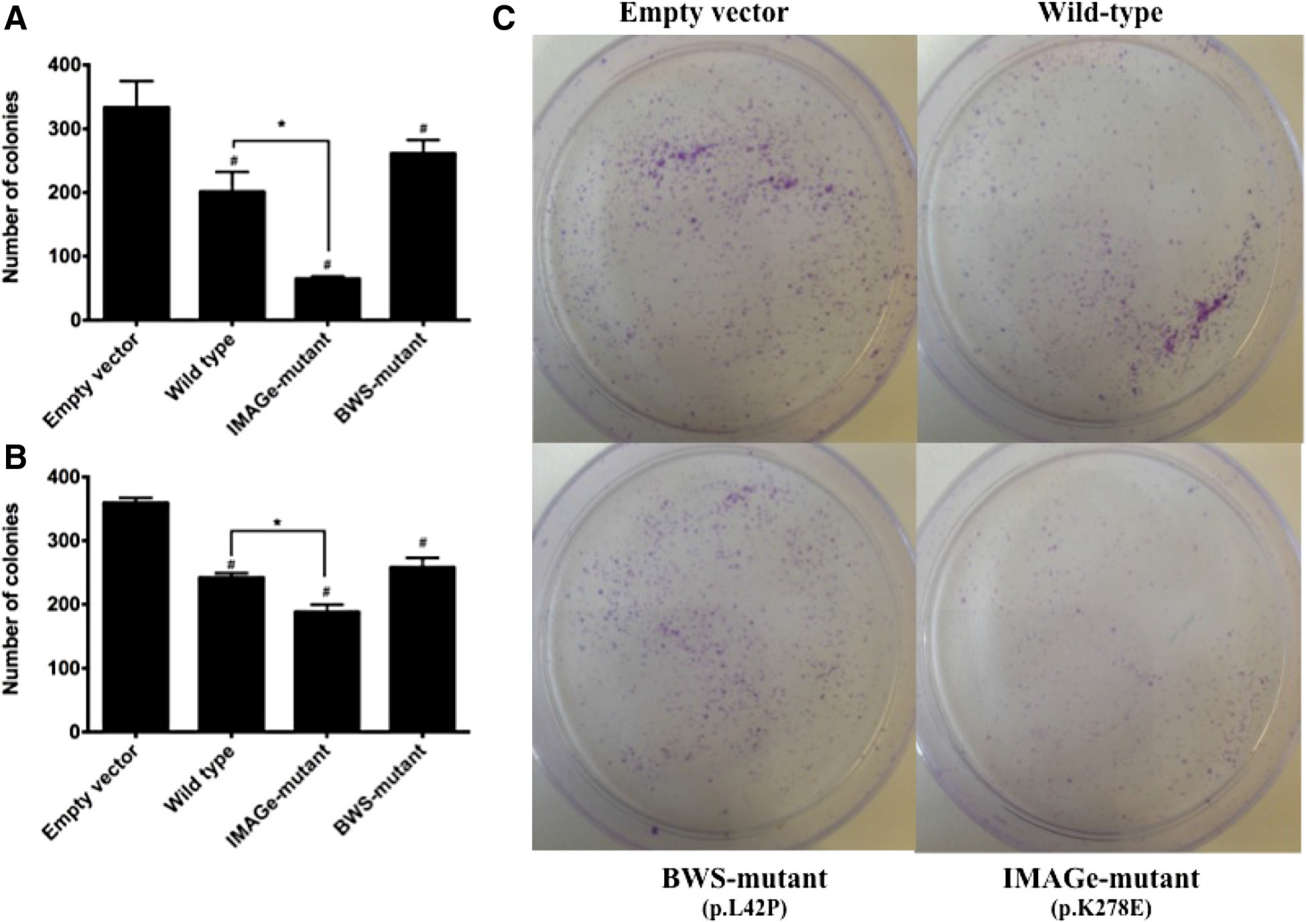
**Clonogenic assay of the HEK293T and SW13 cells transfected with IMAGe-mutant show decreased clonogenic capacity compared to BWS or wild-type CDKN1C**. After transfection of the wild-type CDKN1C, BWS-mutants (p.L42P), IMAGE-mutant (p.K278E) and an empty vector, single cell suspensions were split into six-well in triplicate to assay for clonogenic capacity. **(A)** SW13 and **(B)** HEK293T cells results. # indicates a p < 0.05 when compared to the empty-vector condition. *p = <0.05. **(C)** Representative dishes of SW13 cells transfected as described. Cells were fixed and stained with Crystal violet solution; violet staining represents a higher number of colonies.

All CDKN1C expressing plasmids caused a reduction in the clonogenic capacity (fewer and smaller colonies) when compared to the empty vector in both cell lines (Figure 4A and B). IMAGe-mutant transfected cells displayed even fewer colonies than cells transfected with wild-type CDKN1C in the two cell lines studied (Figure 4A and B).

CDKN1C is a negative regulator of cell proliferation and the effects observed on the wild-type transfected cells may be attributed to a combination of the CDK-inhibitory and PCNA domains of the CDKN1C. Moreover, the remaining effects demonstrated by BWS-mutants probably are associated with the intact PCNA-binding domain of CDKN1C, as described previously [9]. In the IMAGe-mutant transfected cells, the progression through the cell cycle is impaired as cyclical degradation of CDKN1C is lost. The G1-phase cell-cycle inhibition is increased due to increased stability of CDKN1C IMAGe mutants, which maintains an intact cyclin-dependent kinase binding domain, resulting in decreased proliferation and the undergrowth phenotype observed in patients with IMAGe syndrome. Previous reports have described the *in vitro* effects of variations in CDKN1C levels on cell growth [10,11]. *In vivo* models have showed that mouse embryonic growth is sensitive to the precise dosage of *Cdkn1c* and excess of this protein results in dose dependent embryonic growth retardation [12], a phenotype seen in patients with IMAGe Syndrome.

Further expansion of the phenotypes associated with mutations in CDKN1C’s PCNA binding domain suggests phenotypic heterogeneity. Recently, mutations inducing different missense changes of the arginine at amino acid 279 were found in individuals with IMAGe Syndrome and Silver Russell Syndrome (SRS, MIM #180860), characterized by prenatal and postnatal growth retardation and dysmorphic features. We hypothesize that these differences in amino acid changes (arginine to leucine in SRS versus Arginine to Proline in IMAGe Syndrome) are associated with a both differential loss of binding to PCNA and the effects of modifier genes [6]. In addition to SRS, missense mutations located in the highly conserved PCNA binding domain have also been associated with heterogeneous clinical phenotypes with growth restriction and variable to no adrenal failure or skeletal abnormalities and onset of diabetes in early adulthood [13,14]. There the phenotypic spectrum associated with missense mutations in the CDKN1C PCNA binding domain suggests the importance of genetic modifiers in disease manifestation.

Our data suggests that mutations in the PCNA-binding domain of *CDKN1C* leads to increased protein stability, a block in the G1 phase and impaired S-phase entry, and decreased cell proliferation. Our findings demonstrate that domain-specific mutations affect different aspects of cell-cycle progression and cell proliferation, which give rise to the two opposing phenotypes: BWS and IMAGe syndrome. The data presented here give the further direct evidence of how the PCNA-binding site mutations in *CDKN1C* affect the cell cycle and cause IMAGe syndrome.

## Abbreviations

IMAGe: Intrauterine growth restriction, methaphyseal dysplasia, adrenal hypoplasia congenital and genital anomalies
CDKN1C: Cyclin-dependent kinase inhibitor 1C
CDK: Cyclin dependent kinase, BWS, Beckwith Wiedemann syndrome
